# On the Advantages of a Compound Salicylated Plaster

**Published:** 1888-02

**Authors:** 


### *ON THE ADVANTAGES OF A COMPOUND SALICYLATED PLASTER.*

Klotz (New York *Medical Journal*, September 17, 1887), in pursuance of a practice suggested by Prof. Pick, has, for the past several years, used, with great advantage, a salicylated soap plaster. After experimental trials of several formulæ, the following proved the most practical: Emplastri diachyli simplic. emplastri saponati, āā forty parts, petrolati, fifteen parts, acid. salicylici, five parts. The addition of the petroleum ointment was made to render the plaster softer and more pliable. It unfortunately lacks marked adhesive qualities, and requires, therefore, to be kept in place with a bandage. The plaster is inexpensive, one ounce spreading about one square foot. It acts as an antiseptic, forms an efficient protective covering to diseased skin, preventing crust formation; on a dry surface, acting similarly, but in many respects more efficiently, to a rubber bandage, keeping the parts soft and pliable. The plaster finds its widest application in eczema, being useful in every form of this disease except the most acute stages. It acts admirably in fissured eczema of the fingers, in eczema of the forearm and legs; in ulcer of the legs, after healthy granulation has been excited, and, in fact, in granulating wounds resulting from any cause, as burns, dermatitis, caustics, etc. The plaster is changed at intervals of one or several days, according to the necessities ot the case.

